# The mite *Acarus farris* inducing defensive behaviors and reducing fitness of termite *Coptotermes formosanus*: implications for phoresy as a precursor to parasitism

**DOI:** 10.1186/s12862-022-02036-3

**Published:** 2022-06-21

**Authors:** Yong Chen, Lijun Zhang, Shijun Zhang, Bingrong Liu, Wenhui Zeng, Zhiqiang Li

**Affiliations:** 1 Guangdong Key Laboratory of Animal Conservation and Resource Utilization, Guangdong Public Laboratory of Wild Animal Conservation and Utilization, Institute of Zoology, Guangdong Academy of Sciences, Haizhu District, 105 Xingangxi Road, Guangzhou, Guangdong China; 2 Key Laboratory of Plant Protection Resources and Pest Management of Ministry of Education, Entomological Museum, Northwest A&F University, Yangling, China

**Keywords:** Astigmata, Isoptera, Interspecific relationship, Phoresy, Parasitism

## Abstract

**Background:**

The ecology and evolution of phoretic mites and termites have not been well studied. In particular, it is unknown whether the specific relationship between mites and termites is commensal or parasitic. High phoretic mite densities have often been found to occur in weak termite colonies, suggesting that the relationship is closer to that of parasitism than commensalism.

**Results:**

To examine this, *Coptotermes formosanus* was used as a carrier, and *Acarus farris* as the phoretic mite. We used video recordings to observe termite social immunity behaviors and bioassay to examine termite fitness. Our results showed that the attachment of the mite on the termite can enhance termite social immunity behaviors like alarm vibration and grooming frequency while decreasing the duration of individual grooming episodes in phoretic mites. Further, *A. farris* phoresy led to a 22.91% reduction in termite abdomen volume and a 3.31-fold increase in termite mortality.

**Conclusions:**

When termites groom more frequently, the consequence is short duration of grooming bouts. This may be indicative of a trade-off which provides suggestive evidence that frequent social behaviors may cost termites energy. And this caused phoretic behavior hastened termites’ death, and helped propagate the population of mites feeding on dead termites. So, it provides a case for phoresy being a precursor to parasitism, and the specific relationship between *A. farris* and *C. formosanus* is closer to parasitism than to commensalism.

**Supplementary Information:**

The online version contains supplementary material available at 10.1186/s12862-022-02036-3.

## Background

Phoresy, or phoresis, is an interaction in which a phoretic organism (or phoront) attaches itself to a host organism for the purpose of travel [40]. Phoresy is a common form of dispersal behavior throughout Arthropoda [11, 14], for instance, many species of mites disperse by phoresy on social insects like ants, honeybees, and termites [5, 9, 18, 19, 26, 35, 39]. While phoresy is generally recognized to be a commensal interaction in which a phoretic animal is in an inactive/non-feeding stage and thereby does not negatively impact the host [8, 31], recent research has suggested that phoresy can be considered an intermediate precursor to the evolution of parasitism in arthropods [14, 40]. Consequently, this relationship has been viewed as more complex. Existing studies have focused on relationships between mites and honeybees, while there have been few studies on the relationships between phoretic mites and other social insects, or more specifically the evolution of these relationships [25].

Many termite species are pests, destroying houses and wooden structures [12, 34, 38], often living in densely populated colonies in high-humidity habitats [24, 27]. Limited studies on the relationships between mites and termites have been conducted, with most of them being observational in nature [5, 10, 13, 26, 39]. The ecology and evolution of phoretic mites and termites have not been well studied, and it is not known whether the specific relationship between mites and termites is commensal or parasitic in nature. High densities of phoretic mites have often been observed in weak termite colonies and their presence is typically characterized by individuals of the host species exhibiting reduced body weight and a flatter abdomen [30, 39]. Consequently, it is assumed that the relationship in these instances was closer to parasitism than to commensalism.

To investigate the nature of the interspecies relationship between termites and mites, this study observed *Coptotermes formosanus*, a highly destructive termite extant in China, Japan, and the USA, and a representative carrier of social insect mite *Acarus farris*. *A. farris* cause no direct damage to their carrier as the mouthpart is degenerate in the phoretic stage, making it a purely phoretic mite. While *A. farris* is known to be dispersed by the termite *Reticulitermes flavipes* [26], this study represents the first report of *A. farris* dispersal by *C. formosanus*. We aimed to explore whether the interspecies relationship between termites and mites was closer to parasitism than previously assumed. Termites’ social immunity behaviors respond to the presence of some parasites, as they do some pathogens [1, 37, 42]. Using investigations of phoresy based both in the field and laboratory, we observed termites’ social immunity behaviors in the form of grooming and vibration alarm behaviors and fitness costs in terms of abdomen volume and mortality.

## Results

### Termite grooming behavior

Mite phoresy was significantly associated with termite grooming frequency (*χ*^*2*^ = 42.10, *p* < 0.001) and the duration of each grooming episode (*χ*^*2*^ = 20.22, *p* < 0.001).

**Fig. 1:**
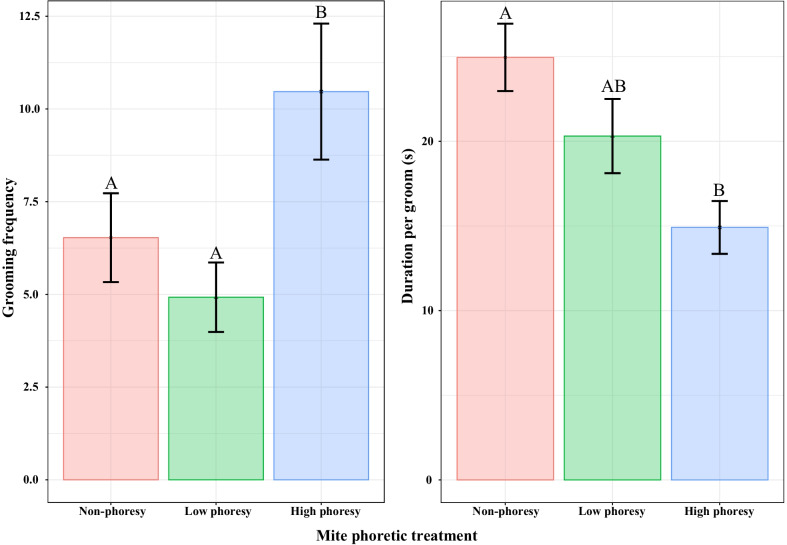
Mean ($\pm$ standard error) grooming frequency and grooming episode duration of termites in different mite phoretic conditions. Capital letters above bars indicate significant differences between phoretic treatments (Tukey’s HSD test p < 0.05)

Termite grooming frequency in the high phoresy group was significantly higher than in the non-phoresy (*z* = 4.19, *p* < 0.001) and low phoresy (*z* = 6.11, *p* < 0.001) groups, by factors of 1.60 and 2.13, respectively. There were no significant differences between the low phoresy and non-phoresy groups (*z* = 2.09, *p* = 0.09) (Fig. 1).

The high phoresy group exhibited significantly shorter duration grooming episodes than did the non-phoresy group (*t* = − 4.35, *p* < 0.001) by 40.26%. There were no significant differences between the high phoresy and low phoresy (*t* = − 2.09, *p* = 0.10), or low phoresy and non-phoresy (*t* = − 1.59, *p* = 0.25) groups (Fig. 1).

### Termite vibration behavior

**Fig. 2:**
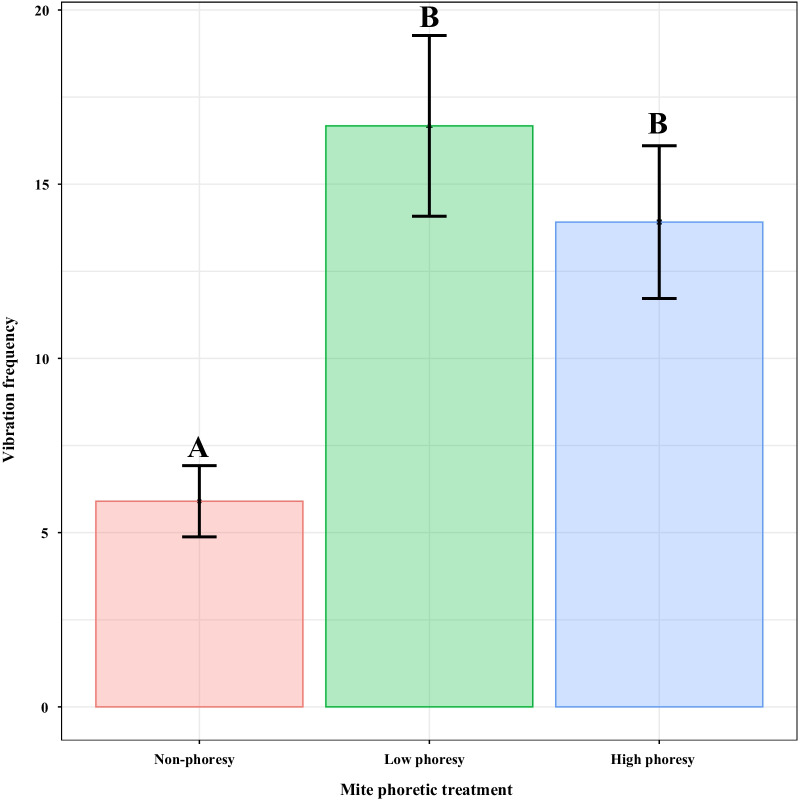
Mean ($\pm$ standard error) vibration frequency of termites in different mite phoretic conditions. Capital letters above bars indicate significant differences between phoretic treatments (Tukey’s HSD test p < 0.05)

Mite phoresy was significantly associated with termite vibration alarm frequency (*χ*^*2*^ = 89.05, *p* < 0.001). Termite vibration alarm frequencies in high (*z* = 7.54, *p* < 0.001) and low (*z* = 9.37, *p* < 0.001) phoresy groups were significantly higher than in the non-phoresy group, by factors of 2.36 and 2.83, respectively. There was no significant difference between the high and low phoresy groups (*z* = 2.15, *p* = 0.079) (Fig. 2).

### Termite abdomen volume and mortality

**Fig. 3:**
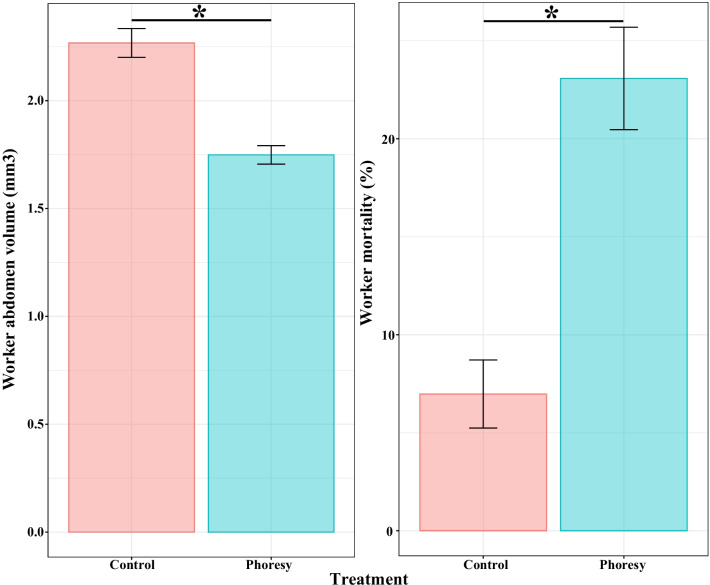
Mean ($\pm$ standard error) worker abdomen volume and mortality. Asterisks indicate a significant difference (α = 5%)

Worker abdomen volume was significantly associated with phoresy group (*χ*^*2*^ = 42.68, *p* < 0.001) (Fig. 3). Compared with the control group (2.27 ± 0.07 mm^3^), the average worker abdomen volume in the phoresy group (1.75 ± 0.04 mm^3^) was 22.91% lower.

Worker mortality was significantly associated with phoresy group (*χ*^*2*^ = 24.64, *p* < 0.001) (Fig. 3). Compared with the control group (6.98 ± 1.74%), the mortality of the phoresy group (23.08 ± 2.61%) increased by a factor of 3.31.

### Mite phoresy in the field and laboratory

**Fig. 4:**
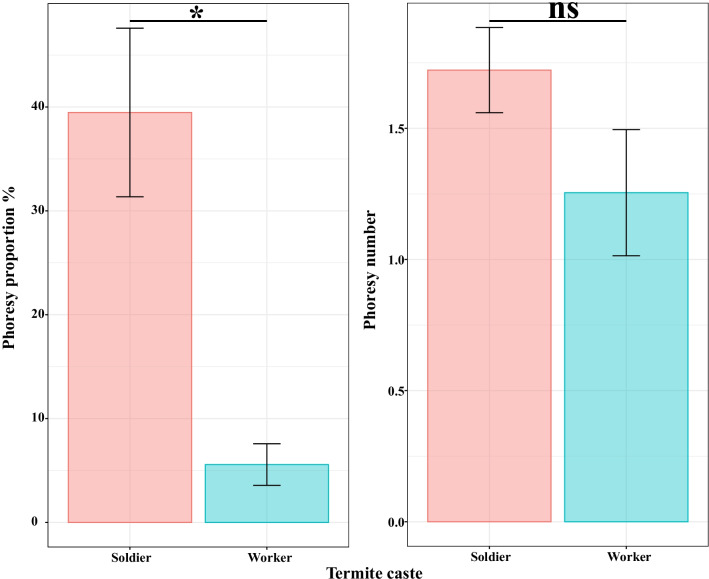
Mean ($\pm$ standard error) phoresy proportion and number in *C. formosanus* in the field. Asterisks indicate a significant difference; ns indicates non-significance (α = 5%)

In the field, the phoresy proportion was significantly associated with termite caste (*χ*^*2*^ = 85.56, *p* < 0.001). The phoresy proportion in soldiers (0.39 ± 0.08) was significantly higher than in workers (0.06 ± 0.02) by a factor of 7.09 (*z* = 9.25, *p* < 0.001). The phoresy number was not significantly affected by termite caste (*χ*^*2*^ = 2.65, *p* = 0.10) (Fig. 4), and was 1.25 ± 0.24 in workers, and 1.72 ± 0.16 in soldiers.

**Fig. 5:**
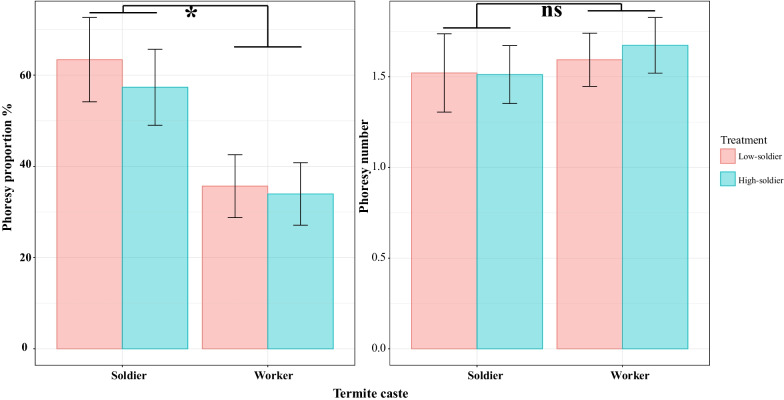
Mean ($\pm$ standard error) phoresy proportion and number in *C. formosanus* in the laboratory. Asterisks indicate a significant difference; ns indicates non-significance (α = 5%)

In laboratory, the phoresy proportion was significantly affected by termite caste (*χ*^*2*^ = 23.92, *p* < 0.001), while not significantly affected by soldier proportion (*χ*^*2*^ = 0.39, *p* = 0.53). The interaction between termite caste and soldier proportion was also non-significant (*χ*^*2*^ = 0.17, *p* = 0.68). The phoresy proportion in soldiers (0.60 ± 0.08) was significantly higher than in workers (0.35 ± 0.06) by a factor of 7.09 (z = 3.36, p < 0.001). The phoresy number was not significantly affected by termite caste (*χ*^*2*^ = 0.58, *p* = 0.45), soldier proportion (*χ*^*2*^ = 0.09, *p* = 0.76) and the interaction was also non-significant (*χ*^*2*^ = 0.07, *p* = 0.80) (Fig. 5). The number of phoretic mites was 1.59 ± 0.15 on workers in the low-soldier condition, 1.67 ± 0.15 on workers in the high-soldier condition, 1.52 ± 0.22 on soldiers in the low-soldier condition, and 1.51 ± 0.16 on soldiers in the high-soldier condition.

## Discussion

In the present study, the social immunity behaviors of *C. formosanus* were enhanced when they were acting as hosts to *A. farris*. Termite grooming and vibration frequency were increased in the higher phoresy group. Termite vibration and grooming behaviors play a key role in social immunity. When termites are exposed to pathogens, they show distinct alarm behaviors, typically consisting of 2–7 s bursts of a rapid longitudinal vibration [6, 26, 33]. Following this, other termites approach and groom exposed nestmates to remove the pathogens from their bodies [42]. Changes in termite social immunity behaviors suggest that termites can recognize their phoretic status and use behavioral responses to remove mites and alert nestmates through high grooming and vibration frequency. The enhanced defensive behaviors were not an immediate state but an ongoing behavior, so we recorded termite behavior after 1 day. And the frequencies of their defensive behaviors were always high. It means the mite attached on termite very tightly and are difficult to remove, so it might not cost much time for the duration of each grooming episode. So, the frequency was increased, while the duration of each groom was decreased.

Phoretic mites show a preference for certain host attachment sites [3, 28, 32]. In the present study, there were only 1–2 mites attached to each termite (Figs. 4, 5.). Further supplementary research (Additional file 1: methods and results) found almost all *A. farris* attached to the head of the termite (Additional file 1: Table S1; Fig S1), and this phenomenon has also been observed among some Acaridae species and termites [39]. The head of *C. formosanus* is therefore considered a suitable site for *A. farris* attachment, and consequently, there is a limited amount of space available for mites to attach to. Differences in head shapes and microstructures between termite castes may lead to mites developing a preference for attaching to specific castes. Soldier termites, with drop-shaped, shiny, harder heads, may be more conducive to mite attachment, as mites are less likely to become dislodged during travel through various environments. Conversely, the oval-ellipsoidal shape and relatively soft texture of a worker’s head may limit the tightness with which mites can attach to the termite, causing them to be more easily removed (Additional file 1: Figs. S2, S3). This may explain the higher phoresy proportion in soldiers than workers (Figs. 4, 5). Further, workers feed themselves, while soldiers are fed by worker trophallaxis, potentially providing mites that attach to soldiers more opportunities for subsequently moving onto other termites.

Phoretic mites in the Acaridae hypopode stage are not generally believed to cause direct damage to the carrier, as their mouthparts are degenerate [15, 22, 29]. The mouthpart of the mite used in the present study is degenerate, and therefore the mite cannot feed on live *C. formosanus*. When frequency increases, duration decreases. This may be indicative of a trade-off, and hence a cost to grooming, i.e., grooming is costly so can only do it in short spurts, and when termites groom more frequently, the consequence is short duration of grooming bouts. Consequently, frequent social immunity behavior may cost higher energy and reduce termite fitness and hasten death. Thompson et al. [37] found termite social contact to be enhanced during exposure to stress, and energy loss was compensated by increasing trophallaxis after removing the stressor. In the present research, the enhanced defensive behaviors were not an immediate state but an ongoing behavior, so we recorded termite behavior after 1 day. And the frequencies of their defensive behaviors were always high. So, termites were under sustained stress, significantly increasing fitness costs may be one of many possible hypotheses and the flat abdomen of termites also provide evidence. The relationship between *A. farris* and *C. formosanus* was, therefore, closer to parasitism than to commensalism, suggestive of the phoretic mite being an intermediate precursor to the evolution of parasitism.

These findings provide direct evidence that mites reduced termite fitness. The findings of previous studies have also reported termite behavior to have been affected by mites, typically with negative effects on termites. Korb and Fuchs [20] reported that individuals were less active in termite colonies where mites were present. In a study by Phillipsen and Coppel [30], the hypopus of *Acotyledon formosani* was found to impede *C. formosanus* feeding. Wang et al. [39] found no significant increase in *Reticulitermes flavipe*s mortality associated with phoresy by the mite *Australhypopus* sp., and the mite was not deemed to be a good candidate for the biological control of termites. The methodology used differed from the present study in several ways. Wang et al. [39] used feeding-stage mites, as opposed to hypopus-stage mites, so the effect observed was not phoretic. Further, while they did not report a significant increase in mortality, they observed that high mite densities occurred in weak termite colonies and that the termites exhibited lower body weights and flatter abdomens. As such, it could be inferred that high mite densities weaken termite colonies. The phoretic mite differs from predatory or typically parasitic organisms in that it indirectly disturbs the termites by attaching itself to their surface. Once the termite dies, the *A. farris* hypopus soon transforms into a tritonymph, a trophic developmental stage, and feeds on the body of the dead termite. Consequently, some mite species may be effective as indirect biological agents to control termite populations.

The present study found that in the field, *A. farris* was commonly attached to *C. formosanus*. In the laboratory, the phenomenon was more clearly observable, with phoresy proportions of 34.81% observed in workers and 60.41% in soldiers. Releasing mites into termite colonies has significant negative effects on termites. Chouvenc et al. [7] suggested that attempting the biological control of termites based only on small-scale laboratory experiments was unrealistically optimistic. However, the biological control agents they referred to were fungi and nematodes, which can be groomed away. The nature of mites is different from that of fungi and nematodes, having a greater potential to control termites as it cannot so easily be groomed away, therefore ensuring that it can bypass the social immunity defenses of termites. Further, *A. farris* can easily be propagated by being fed on cheese, stored rice, and wheat [16, 17, 23, 36], and so could potentially be mass-produced. As such, *A. farris* could provide a useful biological agent for the control of termite populations in specific circumstances such as the protection of ancient buildings and celebrated trees. Further research is necessary to determine its effectiveness and explore how to apply this technique in the field.

## Conclusions

Observations have shown the relationship between *A. farris* and *C. formosanus* to be closer to parasitism than to commensalism. Frequent social immunity behaviors reduced termites’ fitness and enhanced mite populations by providing a food source in the form of dead termites. These energetic costs associated with the presence of *A. farris* are evidence for phoresy being a precursor to parasitism. Further, the use of *A. farris* in populations of *C. formosanus* has the potential to be an effective biological agent in the control of *C. formosanus* colonies to prevent building damage.

## Materials and methods

### Mite and termite collection and rearing

The colony of *C. formosanus* studied had no naturally occurring phoretic mites, was collected using a lure collection device from the South China University of Technology in Guangzhou, China, and was fed on pine. *A. farris* mites were collected from a separate *C. formosanus* colony with phoretic mites, from the South China University of Technology in Guangzhou, China. The presence of dead termites promotes the formation of enormous mite populations. As such, mites were propagated by mixing dead termites (that had been killed by freezing) with healthy termites [39]. Stock colonies of both species were maintained in a greenhouse (temperature 26 ± 1 ℃, 70–80% relative humidity, and a 0 h light/24 h dark photoperiod).

### Termite behavior

To explore termite behavioral responses to phoretic mites, 64 workers were placed into a box containing mites to provide sufficient opportunity for mites to attach themselves to the termites (at a rate of approximately three mites per termite). Control termites comprised 128 workers without mites. Observations were conducted on combinations of host and control termites in 16 replications of each of 3 phoresy conditions: (1) a high phoresy group comprising 3 termites carrying mites and one termite not carrying mites; (2) a low phoresy group comprising one termite carrying mites and 3 termites not carrying mites, and (3) non-phoresy group comprising 4 termites not carrying mites. Each group of 4 termites was placed into a separate 5.5 cm diameter Petri dish padded with a piece of wet filter paper. To observe ongoing behavior, a video camera was used to record termite behavior for 20 min, after 1 day. The grooming frequency, duration of each grooming episode, and vibration (2–7-s bursts of rapid longitudinal oscillatory movement, the grooming and vibration video can be viewed in Additional file 2) frequency of each termite were counted and recorded.

### Termite fitness

To determine the effect of phoresy on termite fitness, two phoresy conditions were compared: (1) a phoresy group comprising 1 soldier and 19 workers carrying mites (using the method described in 2.2), and (2) a control group comprising 1 soldier and 19 workers which were not carrying mites. Each group of termites was kept on wet filter paper in a 15 cm diameter Petri dish. There were 10 replications of each group. After 2 weeks, we counted the number of deceased termites and selected 5 workers randomly that were still alive from each dish. A stereomicroscope (Leica S9i) with bundled software (Leica X) was used to measure termite abdomen length (*L*) and height (*W*). Using the ellipsoid volume formula (*V* = π*L*W^*2*^*/6,* [2], the termite abdomen volume was obtained as an estimate of body size.

### Phoretic mites in the field and laboratory

To explore the presence of mites in field colonies, 6 colonies were investigated in the fall of 2019, in Guangzhou, China. The phoresy proportion was measured using a binary variable to represent whether there was (a value of 1) or was not (a value of 0) a phoretic mite on each termite. The phoresy number represented the number of phoretic mites on each termite. This procedure was conducted for 40 soldiers and 60 workers from each colony.

As soldiers are not naturally numerous in wild colonies, soldier numbers were increased in the laboratory setting. To control for the effect of this increased proportion of soldiers, we compared the effect in laboratory colonies with two different soldier levels: (1) a low soldier group comprising 5 soldiers and 20 workers, and (2) a high soldier group comprising 10 soldiers and 20 workers. To recreate the conditions in the field, 5.0 g soil was placed in 5.5 cm diameter Petri dishes lined with wet filter paper. We placed 2 mites per termite (i.e., 50 mites in the low soldier group and 60 mites in the high soldier group) into each Petri dish. After 1 day, termites were transferred to the dishes. After 7 further days, the phoresy proportion and number in each dish were recorded. There were 10 replications of each group.

### Data analysis

A generalized linear mixed-effects model was used to investigate the phoresy number (Poisson error structure, log link function) and phoresy proportion (binomial error structure, log link function) in the field and the laboratory, with colonies used as random intercepts in the field and repeats used as random intercepts in the laboratory. The duration of each grooming episode was investigated using a general linear mixed-effects model, with group replications as random intercepts. Grooming frequency and vibration alarm frequency were investigated using generalized linear models (Poisson error structure, log link function). Termite mortality was investigated using a further generalized linear model (binomial error structure, log link function). Finally, termite abdomen volume was investigated using a general linear model, with the individual termites in each dish used as random intercepts. The R package lme4 was used to construct the mixed-effected models [4]. Visual inspection of residual plots did not reveal any obvious deviations from homoscedasticity or normality. After detecting a significant effect, the Tukey posthoc comparison procedure was used to make pairwise comparisons between treatments using the emmeans R package [21]. All data analyzes were conducted using R 4.0.2 statistical software (R Development Core Team, 2020), and figures were created using the R package ggplot2 [41].

## Supplementary Information

**Additional file 1:** **Table S1.** Analysis of Deviance Table (Type II Wald chi-square tests). **Fig S1.** Mean (±SE) phoresy proportion in different part of *C. formosanus.* **Fig S2.** Mean (±SE) proportion and number of phoresy on two castes of *C. formosanus.* **Fig S3.** Location of *A. farris *attachment on the head of two *C. formosanus* castes (left: soldier; right: worker; red arrow: mite).

**Additional file 2:** Video of vibration and grooming of termites.

## Data Availability

The datasets used and/or analysed during the current study are available from the corresponding author on reasonable request.
