# Supplementary material for: The mite Acarus farris inducing defensive behaviors and reducing fitness of termite Coptotermes formosanus: implications for phoresy as a precursor to parasitism
Source: BMC Ecol Evol. 2022 Jun 21;22:80. doi: 10.1186/s12862-022-02036-3 (PMC9210751; doi:10.1186/s12862-022-02036-3)
Supplement: Supplementary file 1 — Additional file 1: Table S1. Analysis of Deviance Table (Type II Wald chi-square tests). Fig S1. Mean (±SE) phoresy proportion in different part of C. formosanus. Fig S2. Mean (±SE) proportion and number of phoresy on two castes of C. formosanus. Fig S3. Location of A. farris attachment on the head of two C. formosanus castes (left: soldier; right: worker; red arrow: mite). [file 12862_2022_2036_MOESM1_ESM.doc]

[Supplementary](javascript:;) [trial](javascript:;)

**Method:** To determine the barrier environment whether affects the tightness of the attachment between the mites and termites, we chose one termite colony in field which with a very small phoresy proportion compared with the other colonies to conduct these experiments. We set two block environment: 1) Unimpeded: 6-cm diameter [Petri](../../../../F:/%25E5%2590%2584%25E8%25BD%25AF%25E4%25BB%25B6%25E5%258E%259F%25E5%25A7%258B%25E5%25AE%2589%25E8%25A3%2585%25E5%258C%2585/Youdao/Dict/8.8.1.0/resultui/html/index.html#/javascript:;) [dish](../../../../F:/%25E5%2590%2584%25E8%25BD%25AF%25E4%25BB%25B6%25E5%258E%259F%25E5%25A7%258B%25E5%25AE%2589%25E8%25A3%2585%25E5%258C%2585/Youdao/Dict/8.8.1.0/resultui/html/index.html#/javascript:;) which with a piece of wet filter paper, and termites can eat paper unimpeded; 2) Impeded: soil in 5 cm diameter pot which with a piece of wet pine, and termites had to move through soil for eating. We placed 60 mites into each [Petri](../../../../F:/%25E5%2590%2584%25E8%25BD%25AF%25E4%25BB%25B6%25E5%258E%259F%25E5%25A7%258B%25E5%25AE%2589%25E8%25A3%2585%25E5%258C%2585/Youdao/Dict/8.8.1.0/resultui/html/index.html#/javascript:;) [dish](../../../../F:/%25E5%2590%2584%25E8%25BD%25AF%25E4%25BB%25B6%25E5%258E%259F%25E5%25A7%258B%25E5%25AE%2589%25E8%25A3%2585%25E5%258C%2585/Youdao/Dict/8.8.1.0/resultui/html/index.html#/javascript:;). After 1 day, we transferred 5 soldiers and 20 workers to the corresponding environments, and, after 7 days, we counted phoresy proportion and number for each replicate experiment. At the same time, we also counted phoresy proportion in different part of termite body surface (left head, right head and body) to determine the mites whether show a preference for the attachment site on their carrier. There were 10 replicates for each treatment.

**Result:** All two or three interaction of termite caste, environmental condition and termite body part are not significantly affected mites location choice (Table S1.). So, we only considered on the independent action of the three variable. There was a significant differences among termite body parts (*χ^2^* = 54.64, *p* < 0.01), while was no significant differences between termite castes (*χ^2^* = 1.09, *p* = 0.30), environmental conditions (*χ^2^* = 0.47, *p* = 0.49) (Fig S1.). The proportion of mites located on termite left head and right head was higher than on body by 29.24-fold (*z* = 3.29, *p* = 0.003) and 24.07-fold (*z* = 3.03, *p* = 0.007), respectively. And there was not significantly difference between left head and right head (*z* = 1.55, *p* = 0.27).

The phoresy proportion was significantly affected with environmental condition (*χ^2^* = 26.37, *p* < 0.001), the interaction of environment condition and termite castes (*χ^2^* = 30.95, *p* < 0.001), while not by termite castes (*χ^2^* = 0.02, *p* = 0.90) (Fig S2.). In Impeded environment, the proportion in soldiers (0.62±0.07) was significantly higher than in workers (0.38±0.04) by 1.63 times (*z* = 3.17, *p* = 0.002). In Unimpeded environment, the proportion in soldiers (0.37±0.09) was significantly lower than in workers (0.82±0.04) by 2.22 times (*z* = -4.60, *p* < 0.001).

The phoresy number was significantly affected by environmental condition (*χ^2^* = 4.39, *p* = 0.04), while not by termite castes (*χ^2^* = 0.93, *p* = 0.33) and the interaction of the two (*χ^2^* = 0.99, *p* = 0.32) (Fig S2.). The number in Unimpeded environment (1.90±0.16) was significantly higher than in Impeded environment (1.53±0.12) by 1.24 times (*z* = 2.10, *p* = 0.036).

**Table S1. Analysis of Deviance Table (Type II Wald chi-square tests)**

| Factor | *χ^2^* | Df | *p* ( > *χ^2^*) |
| --- | --- | --- | --- |
| Caste | 0.83 | 1 | 0.36 |
| Body part | 42.11 | 2 | < 0.001 |
| Environmental condition | 1.37 | 1 | 0.24 |
| Caste*Body part | 2.85 | 1 | 0.24 |
| Caste*Environmental condition | 0.16 | 1 | 0.69 |
| Body part*Environmental condition | 0.42 | 2 | 0.81 |
| Caste*Body part*Environmental condition | 3.03 | 2 | 0.22 |


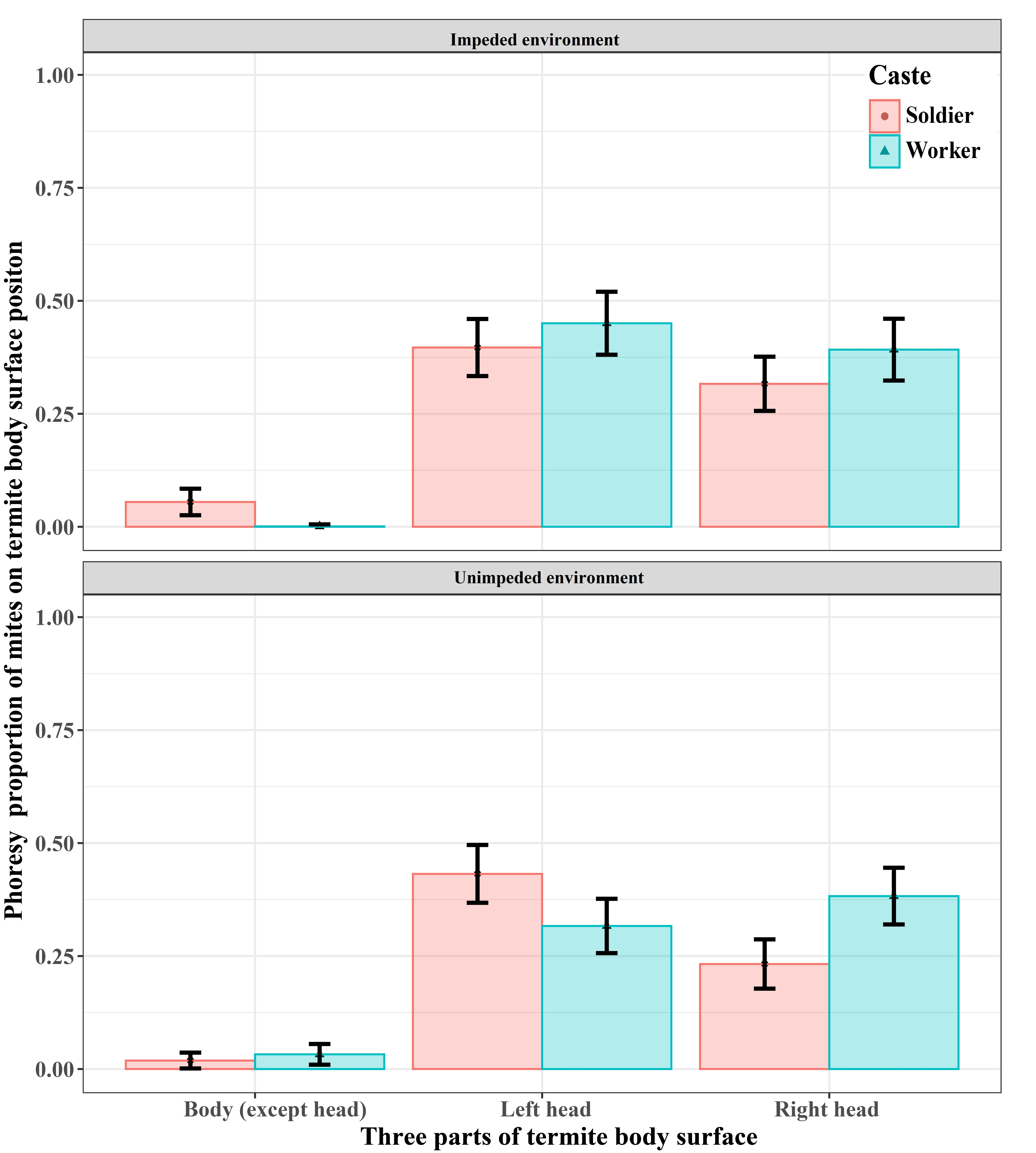


**Fig S1. Mean ($\pm$SE) phoresy proportion in different part of *C. formosanus***


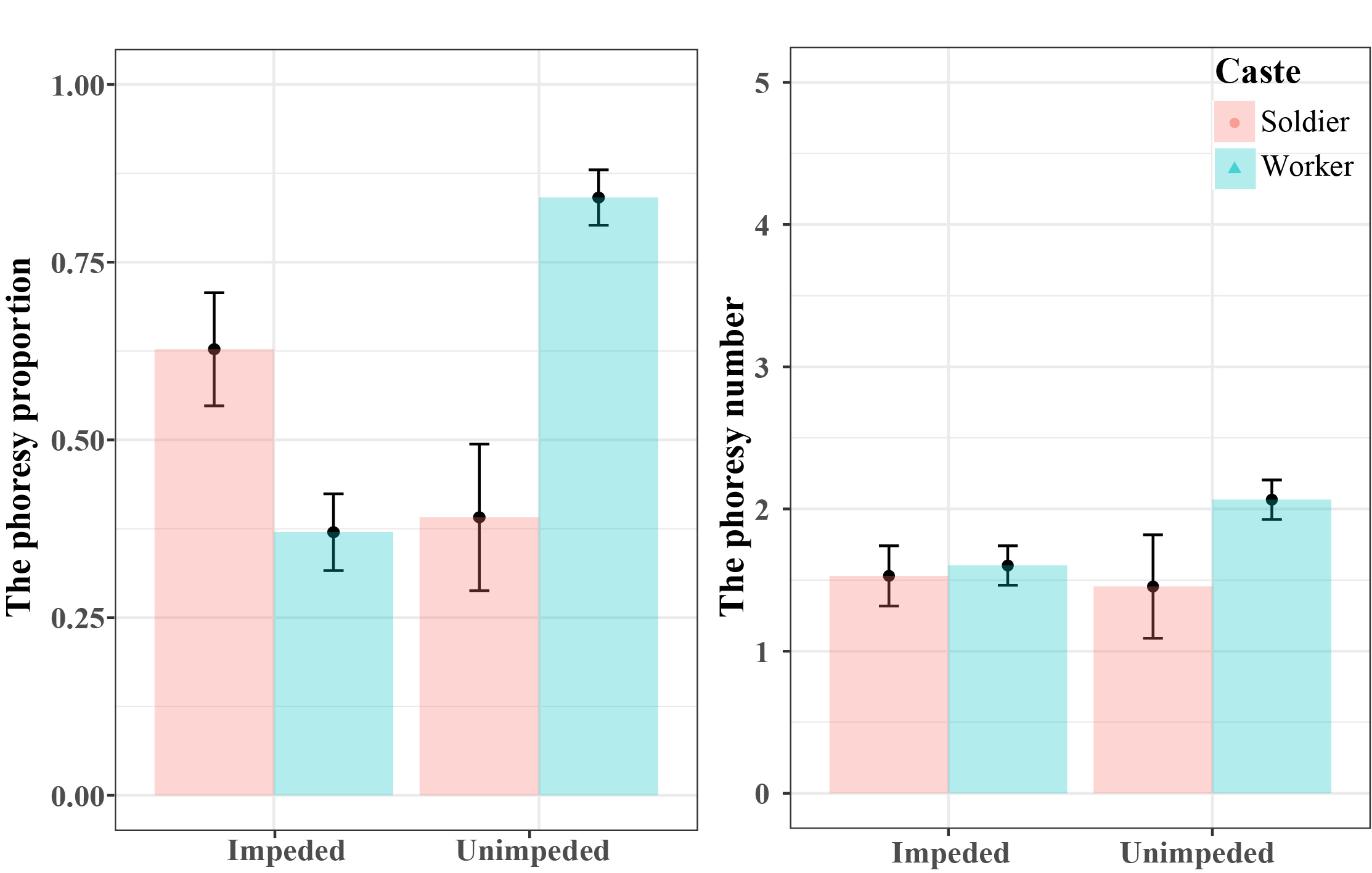


**Fig S2. Mean ($\pm$SE) proportion and number of phoresy on two castes of** ***C. formosanus***

***
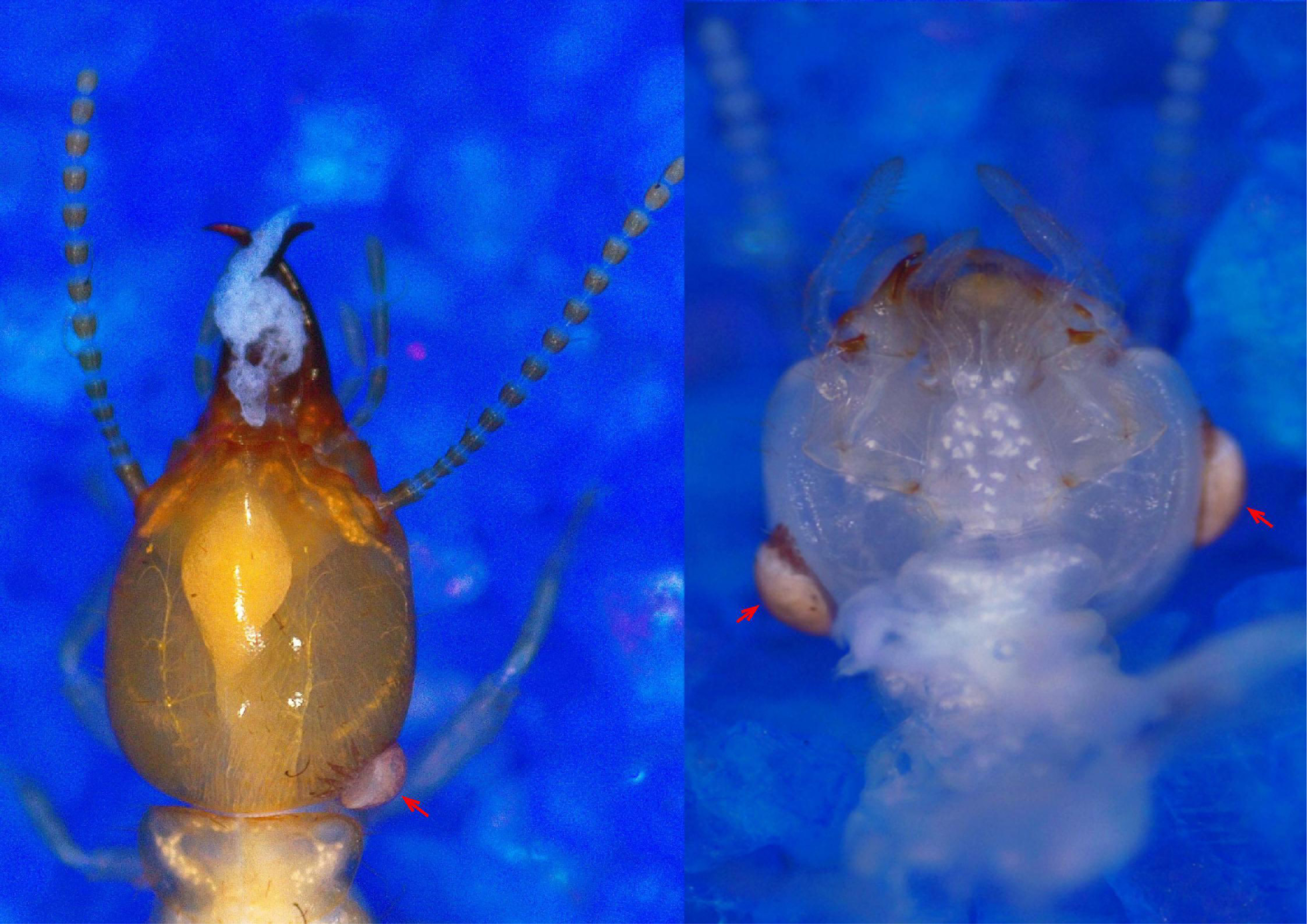
***

**Fig S3. Location of *A. farris* attachment on the head of two *C. formosanus* castes** (left: soldier; right: worker; red arrow: mite)
